# Smartphone Heading Correction Based on Gravity Assisted and Middle Time Simulated-Zero Velocity Update Method

**DOI:** 10.3390/s18103349

**Published:** 2018-10-07

**Authors:** Qinghua Zeng, Shijie Zeng, Jianye Liu, Qian Meng, Ruizhi Chen, Heze Huang

**Affiliations:** 1 Navigation Research Center (NRC), Nanjing University of Aeronautics and Astronautics (NUAA), Nanjing 210016, China; zengshijie@nuaa.edu.cn (S.Z.); ljyac@nuaa.edu.cn (J.L.); mengqian@nuaa.edu.cn (Q.M.); nrchhz@nuaa.edu.cn (H.H.); 2 State Key Laboratory of Information Engineering in Surveying, Mapping and Remote Sensing, Wuhan University, Wuhan 430079, China; ruizhi.chen@whu.edu.cn

**Keywords:** gravity assisted, simulate-zero velocity update, smartphone navigation, dead reckoning

## Abstract

Electronic appliances and ferromagnetic materials can be easily found in any building in urban environment. A steady magnetic environment and a pure value of geomagnetic field for calculating the heading of the smartphone in case of pedestrian walking indoors is hard to obtain. Therefore, an independent inertial heading correction algorithm without involving magnetic field but only making full use of the embedded Micro-Electro-Mechanical System (MEMS) Inertial measurement unit (IMU) device in the smartphone is presented in this paper. Aiming at the strict navigation requirements of pedestrian smartphone positioning, the algorithm focused in this paper consists of Gravity Assisted (GA) and Middle Time Simulated-Zero Velocity Update (MTS-ZUPT) methods. With the help of GA method, the different using-mode of the smartphone can be judged based on the data from the gravity sensor of smartphone. Since there is no zero-velocity status for handheld smartphone, the MTS-ZUPT algorithm is proposed based on the idea of Zero Velocity Update (ZUPT) algorithm. A Kalman Filtering algorithm is used to restrain the heading divergence at the middle moment of two steps. The walking experimental results indicate that the MTS-ZUPT algorithm can effectively restrain the heading error diffusion without the assistance of geomagnetic heading. When the MTS-ZUPT method was integrated with GA method, the smartphone navigation system can autonomously judge the using-mode and compensate the heading errors. The pedestrian positioning accuracy is significantly improved and the walking error is only 1.4% to 2.0% of the walking distance in using-mode experiments of the smartphone.

## 1. Introduction

Location Based Service (LBS), also known as mobile location information service, is a widely used service which can determine the actual geographical location of users and provide users with the location-related information at any time in order to meet the requirements of different users [1,2]. With the continuous development of smartphone technology, smartphone-based pedestrian navigation has become an important emerging branch in the field of pedestrian navigation, and it has broad prospects for kinds of applications [3]. For instance, smartphone can be utilized to determine and monitor the location and movement of pedestrian. In the event of an unexpected disaster, the positioning information provided by the smartphone can greatly improve the rescue efficiency of government. Smartphone navigation can also be applied to ensure the safety of pedestrian activities. Aim at this research trend in the field of navigation, universities and research institutes around the world have conducted multitudinous researches in LBS. In 2017, the National Institute of Standards and Technology (NIST) of the U.S. Department of Commerce, held a Grand Prix called “PerfLoc”, to encourage participants to develop better algorithms for indoor location and tracking.

At present, the pedestrian positioning and navigation methods in urban environment mainly focus on satellite navigation and its integrated navigations, such as GNSS (Global Navigation Satellite System) and INS (Inertial Navigation System) integrated system. When pedestrian is in an outdoor environment, satellite navigation is the most important tool, and the research on satellite navigation in recent years has also achieved an appreciable performance [4]. However, modern lifestyle makes it possible for pedestrians to study, work, play in an indoor environment for almost the entire day. As for indoor environments, navigation research mainly focuses on two kinds of methods. The first one is the radio frequency devices, such as UWB (Ultra-Wideband), ZigBee, wireless networks, Wi-Fi, Bluetooth. Another one is the inertial sensors or IMU, which are mounted on human leg, waist, shoulder or other parts [5]. Inertial navigation results can be achieved with the help of ZUPT and Pedestrian Dead Reckoning (PDR) algorithms [6]. The two kinds of navigation methods mentioned above have both merits and demerits. The first navigation method needs to deploy radio frequency devices as beacons before commencing navigation, which increases the costs of the system. While inertial navigation algorithm, such as PDR algorithm, is an autonomous navigation without contacting the external environment, has become the primary part of pedestrian navigation system. However, inertial navigation algorithm still has some disadvantages, the most important research content for INS is to slow down its divergence speed. If we use the PDR algorithm only, it is very difficult to obtain the absolute position, due to the limitations of the PDR algorithm. Additional navigation equipment or information are needed to amend INS results. And recently, the integrated navigation methods, such as INS/UWB, INS/WIFI and INS/Bluetooth [7,8,9], have been widely used. For instance, Diaz proposed a method based on the angle measurement to discriminate the step, attitude, and step-length to optimize pedestrian inertial navigation results [10]. Zam used magnetometers, gyroscopes and accelerometers in conjunction with Extended Kalman Filtering (EKF) to obtain more accurate heading information for the PDR algorithm [11]. These integrated navigation algorithms show good navigation results, but still require extra navigation information. Once users are in an unfamiliar building without any radio frequency equipment installed, the effect of these integrated algorithms will be greatly reduced. 

As a portable communication device, smartphone has been studied and improved in recent years. Lots of sensors have been integrated inside the smartphone, including accelerometer, gravimeter, gyroscope, magnetometer, and so on. The smartphone has become a great platform to study pedestrian navigation. Zeng and his team designed and implemented a seamless indoor and outdoor navigation system based on a smartphone [12]. Broyles presented a real-time, self-contained outdoor navigation application, only used the existing sensors on a smartphone in conjunction with a preloaded digital elevation map [13]. Li proposed an algorithm for navigation with smartphone sensors and magnetic matching in Non-WiFi indoor environment [14]. In the inertial navigation research based on the smartphone, Valerie designed a hybridization filter to calibrate the PDR step-length [15]. Kang put forward and realized a smartphone-based algorithm SmartPDR navigation system, which can be used in the smartphone with its PDR navigation solution [16].

When the PDR algorithm is used for smartphone navigation by a pedestrian indoors, it is very hard to keep smartphone stable. At this time, the using-mode of smartphone, or the smartphone’s handheld posture must be paid a particular attention. Generally, pedestrian has to keep the smartphone fixed relative to his body in order to reduce different using-mode errors on pedestrian navigation while walking indoors. But in the daily life, it is hard for user to hold the smartphone with one fixed posture, and the indoor complex magnetic environment makes it impossible to correct the heading angle directly with magnetic field. Therefore, in this paper, an algorithm which does not depend on the environment of a magnetic field has been proposed. This algorithm based on the smartphone’s self-contained MEMS inertial sensors, can discriminate the smartphone’s using-mode and handheld posture by analyzing the output of MEMS sensors, and it can compensate the heading error caused by three different using-modes.

## 2. Principle of Pedestrian Dead Reckoning

The PDR algorithm is a continuous and real-time positioning method based on step characteristics of pedestrian, which can achieve better navigation accuracy with low cost devices. The features of PDR algorithm, such as complete autonomy and flexibility, make it popular in the pedestrian navigation field. PDR algorithm is comprised of three important parts [17,18]:

(1) Step Detection;
(2) Step Length Estimation;
(3) Heading Estimation.

**Figure 1:**
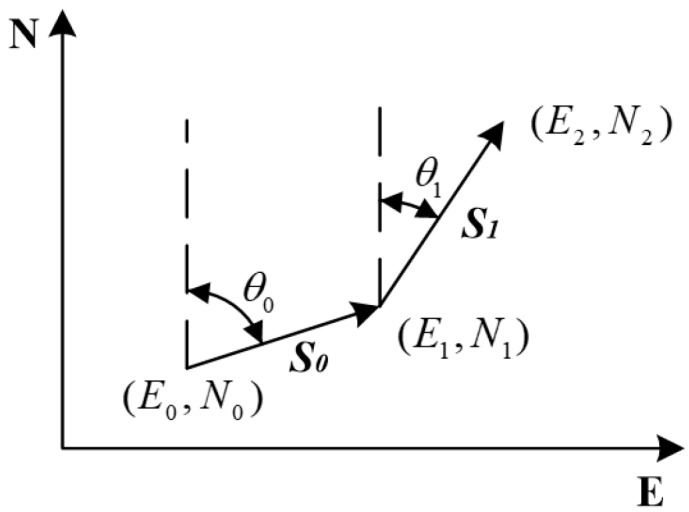
Simple Schematic Diagram of PDR Algorithm.

Figure 1 is a simple schematic diagram of the PDR algorithm. 

In this Figure, *N* and *E* represent the North and East directions respectively. ($E_{i},N_{i}$) is the pedestrian positioning coordinate. $\theta_{i}$ represents the angle between pedestrian forward direction and North direction.

(1) $$\;E_{k}=E_{0}+\overset{k-1}{\underset{i=0}{\sum }}S_{i}\sin\theta_{i}\;\;N_{k}=N_{0}+\overset{k-1}{\underset{i=0}{\sum }}S_{i}\cos\theta_{i}\;$$

In this formula, $E_{0}$ and $N_{0}$ are the initial east and north coordinates in the northeast coordinate system. $E_{k}$ and $N_{k}$ are the east and north coordinates of step *k*. $\theta_{i}$ is the angle between pedestrian forward direction and the North direction of step *i*. 

## 3. Heading Correction Algorithm Based on Middle Time Simulated-Zero Velocity Update

The accuracy of the PDR algorithm is greatly influenced by the sensor. The low-cost gyroscope and accelerometer make it impossible to get high precision positioning results. ZUPT, a simple but reliable method of inertial navigation error compensation, can purposefully reduce the error accumulation in inertial navigation system and significantly boost the positioning accuracy of inertial navigation [19], so it has been widely used in pedestrian navigation to lessen the heading divergence caused by low-precision sensor’s drift. The most important part of the ZUPT algorithm is the detection of stationary state, then the outputs of the sensors at this stationary state are put into Kalman Filter (KF) as the system errors. After the filtering process, the optimal estimation result is used to correct the system errors [20].

Because the zero-velocity status can be distinguished by the output of accelerometer and gyroscope when foot is touching ground, the inertial sensors are normally fixed to the foot of the experimenter while using ZUPT as heading compensation in an integrated navigation algorithm. However, in this paper, we use the smartphone to collect the experiment data. Considering the shape, size as well as the using methods of smartphone, the smartphone is held in hands during the experiments, instead of foot mounted. At this time, the smartphone cannot detect the real zero-velocity status, which makes it impossible to use the traditional ZUPT algorithm. In this paper, we propose a Simulated-Zero Velocity Update(S-ZUPT) algorithm to improve the navigation process.

First, a step-detecting algorithm is designed. The signal output from the three-axis accelerometer is pre-processed as follows:

(2) $$\;a=\sqrt[{2}]{a_{x}^{2}+a_{y}^{2}+a_{z}^{2}}\;$$

where $a_{x}$, $a_{y}$ and $a_{z}$ are the 3D acceleration information of the smartphone, a represents the sum of the three-axis acceleration information. Commonly, there are three methods to detect steps, Peak Detection, Average Interval Detection and Zero-Crossing Detection [21]. In order to reduce the computational complexity, we choose Peak Detection as step-detecting method.

Due to the low-cost sensor and walking swing, the peak points of the true pace are affected by noise. To detect the real steps, two constraints are added to develop the accuracy of step detection:

(1) The acceleration peak must be greater than a certain threshold to avoid false peak caused by other vibrations;
(2) The time interval ∆T between two consecutive peaks must be limited in the thresholds, which is helpful to remove the multi-peak effect.

Considering the above two constraints in mathematical form, we have

(3) $$\;\{\begin{array}{c} a>A\\ t_{min}<∆T<t_{max} \end{array}\;$$

where A is the acceleration peak threshold, $t_{min}$ and $t_{max}$ represent the minimum and maximum value of the time intervals. Considering the walking frequency of pedestrians is generally between 2 Hz and 4 Hz, we set $t_{max}=0.5\;s$ and $\;t_{min}=0.25\;s$.

Several experiments have been conducted to validate the algorithm, and the results are shown in Table 1. From the Table 1, we can see that in all of the four experimental results, the step number is only one or two steps less than the actual number. The accuracy of single experiment can reach more than 98.3% and the average accuracy is 99.2%.

**Figure 2:**
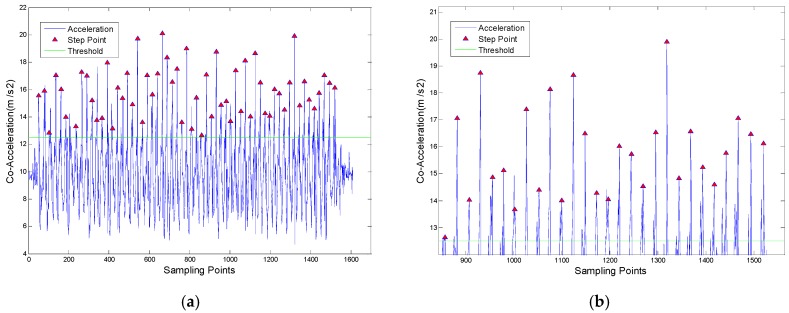
Experiment Results of Peak Detection Algorithm. (**a**) Overall Step Detection Results; (**b**) Step detection result of partial enlargement.

In the Figure 2, the red triangle shows the detected peak point. From it we can judge that the value of each step peak point is different, but the sampling interval between steps is similar. The low accuracy of the smartphone sensor makes the different peak values, while the stable pedestrian walking law makes the sampling interval of steps to remain consistent.

**Figure 3:**
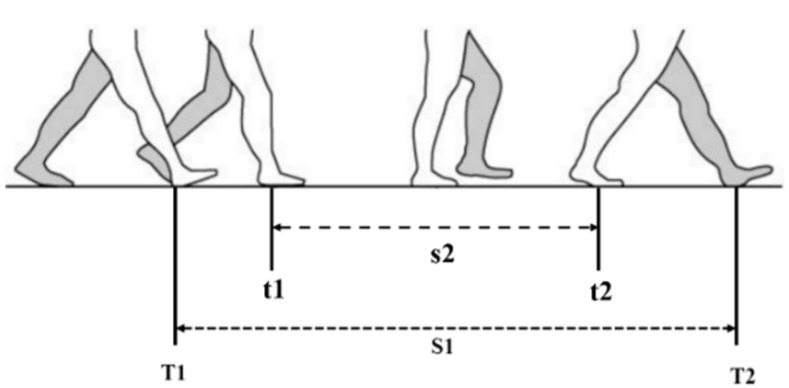
A Typical Step Cycle.

Since there is no traditional zero-velocity status for handheld smartphone navigation, choosing a right method for KF is very important. A typical step cycle is shown in Figure 3. S1 between the right foot heel landing at T1 and the left foot heel landing at T2 is a complete step cycle. T1 and T2 are defined as step time. The process s2 between t1 and t2 represents the period when pedestrian lifts one foot in the air. In the study of pedestrian’s walking status, we found that the turning and heading changes of the pedestrian happen in s2 part. According to Newtonian Mechanics, another force is required to change the motion of an object while moving. In s2 process, the right foot supports the human body to change his heading. During this time, the heading change of smartphone is consistent with that of the human body. At same time, a Simulated-Zero Velocity Update (S-ZUPT) status, similar as the traditional ZUPT status, was found within s2.

According to the Step Cycle, two kinds of S-ZUPT algorithms are proposed to realize in this paper. If the KF algorithm is applied at the middle of the two detected steps, we call it Middle Time Simulated-Zero Velocity Update (MTS-ZUPT). Corresponding to the MTS-ZUPT, using KF algorithm at step time is called Step Time Simulated-Zero Velocity Update (STS-ZUPT). We define $\frac{T_{1}+T_{2}}{2}$ as Middle-time, and start filtering at the Middle-time instead of step time. The characteristics of human motion were used to construct observation model. The system and measurement formulas of the filtering system are set as following:

(4) $$\;\{\begin{array}{c} \dot{X}\left(t\right)=F\left(t\right)X\left(t\right)+G\left(t\right)w\left(t\right)\\ Z\left(t\right)=H\left(t\right)X\left(t\right)+v\left(t\right) \end{array}\;$$

In Formula (4), *F*(*t*) is the system state matrix, *H*(*t*) is the system observation matrix, *G*(*t*) is the system noise matrix, *w*(*t*) is the system noise, and *v*(*t*) is the observed noise. After discretization solution, we can get the system discrete state estimation equation and system observation equation at time *k* + 1.

(5) $$\;{\hat{X}}_{k+1}=\;F_{k}{\hat{X}}_{k}+\;G_{k}W_{k}\;$$

where $F_{k}=\left[\begin{array}{cc} I & 0_{3\times 3}\\ 0_{3\times 3} & ∆t\times I \end{array}\right]$ is the transfer matrix, *I* is a third-order unit matrix, and ∆t is sampling time. $G_{k}$ is system noise matrix, and $W_{k}$ is the system noise.

The state quantity of the Kalman filter ${\hat{X}}_{k}$ consists of six parameters in the ENU (East, North and Up, ENU) coordinate system:

(6) $$\;{\hat{X}}_{k}=\;{\left[y_{k}\;\theta_{k}\;\varphi_{k}\;\omega_{xk}\;\omega_{yk}\;\omega_{zk}\;\right]}^{T}\;$$

where *k* is one of the Middle-time. And $\;y_{k},\theta_{k},\varphi_{k},\omega_{xk},\omega_{yk},\omega_{zk}$ are the roll, pitch and heading angle and the angular velocity values of the three axes of gyroscope at that moment.

The horizontal attitude angle and heading angle, which are obtained by the pedestrian dead reckoning system, and the real-time gyro output angular velocity value are taken as the observation of the system. The observation equation at *k* + 1 moment can be obtained as follows:

(7) $$\;Z_{k+1}=\;H{\hat{X}}_{k}+Bu_{k+1}\;$$

where $H=\;\left[\begin{array}{cc} I & 0_{3\times 3}\\ 0_{3\times 3} & 0_{3\times 3} \end{array}\right]$ and $B=\left[\begin{array}{c} ∆t\times I\\ I \end{array}\right]$, $u_{k+1}$ is the real time angular velocity of gyroscopes, the γ, θ and φ are pitch, roll and heading angles separately, which can be derived from the pedestrian dead reckoning system.

Limited by the characteristics of pedestrian walking, the heading angle output period is non-uniform. Based on the analysis of the periodic characteristics of the pedestrian’s steps, the time-varying discrete heading angle output period is selected:

(8) $$\;\{\begin{array}{c} T_{D}=t-old_{t}\\ old_{t}=t \end{array}\;$$

In the iterative relationship of the above formula, $T_{D}$ is the heading angle output period, $old_{t}$ is the previous heading angle output time point, which is the last Middle-time; t is the current Middle-time.

The information of attitude angle and angular velocity information calculated by the MTS-ZUPT algorithm are the optimal estimation values for each Middle-time.

## 4. Gravity Assisted Method 

**Figure 4:**
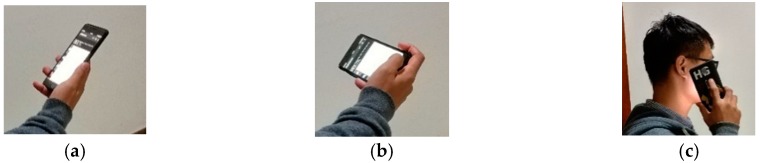
Three Using-modes of Smartphone. (**a**) Normal mode as Mode 1; (**b**) Landscape mode as Mode 2; (**c**) Call mode as Mode 3.

In the normal routine, we have various using-modes to carry the smartphone, such as answering the phone call, surfing the web, or even rotating the phone to landscape mode, as shown in the Figure 4. Each mode has its special posture. For the convenience of description, we use Mode 1, Mode 2 and Mode 3 to denote the normal mode, landscape mode, and call mode, respectively.

When one using-mode switches to another using-mode in Figure 4, there will be large attitude changes. The value of acceleration and angular rate during the process of attitude changing will be detected and recorded by the smartphone sensors, which will mislead the traditional PDR navigation system, and the heading of pedestrian will be changed to a wrong value. In this paper, after analyzing the using-modes of smartphone and the corresponding sensors output, a Gravity Assisted (GA) algorithm is proposed to compensate the heading of pedestrian navigation.

**Figure 5:**
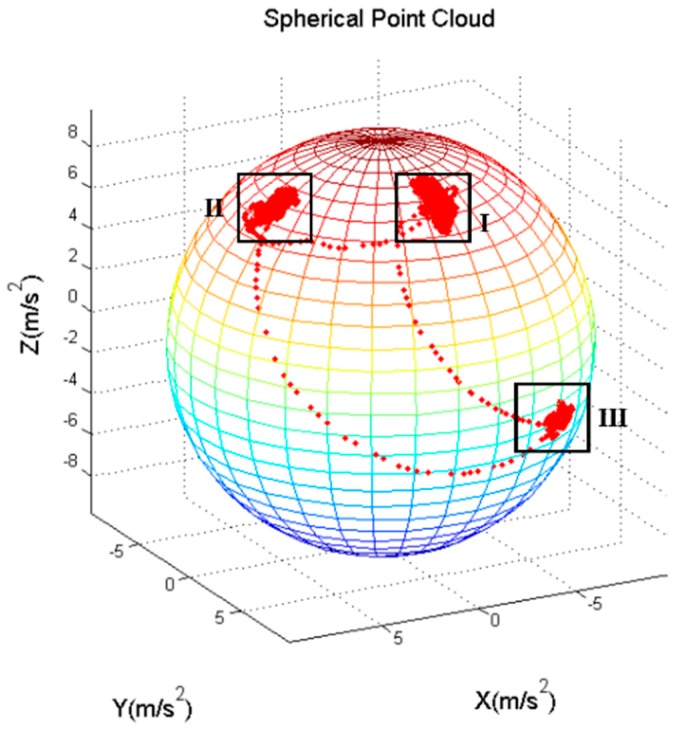
Spherical Point Cloud.

The GA algorithm is divided into two parts, the attitude discrimination method and the heading compensation process. The gravity data detected by gravimeter of smartphone has the following features:

(9) $$\;\vec{g}={\vec{g}}_{x}\;+\;{\vec{g}}_{y}+\;{\vec{g}}_{z}\;$$

where $\vec{g}$ means earth gravity vector, ${\vec{g}}_{x}\;,\;{\vec{g}}_{y},\;{\vec{g}}_{z}$ are the gravitational acceleration vector components of *x*, *y*, and *z* axes of the smartphone. If the three-axis gravimeter data are projected into a three-dimensional coordinate system, a sphere can be realized, and a spherical point cloud can be seen in Figure 5. In this Figure, each point represents the data of a sampling point. And the radius of the sphere is the local gravitational acceleration value. In addition, area I, II, III represent the gravity data projection of Mode 1, Mode 2 and Mode 3 respectively. From Figure 5, we can find that the gravitational output data in different mode is projected in different areas, which means that the using-mode of the smartphone can be distinguished, by calculating the azimuth and elevation angle with the gravimeter data.

Formula (10) shows how to calculate the azimuth and elevation angles by gravity data. $g_{x},{\;g}_{y},{\;g}_{z}$ are three output of axis gravimeters, and α, β are azimuth and elevation angles.

(10) $$\;\{\begin{array}{c} \alpha =\arctan\left(\frac{g_{y}}{g_{x}}\right)\\ \beta =\arccos\left(\frac{g_{z}}{\sqrt{g_{x}^{2}+g_{y}^{2}+g_{z}^{2}}}\right) \end{array}\;$$

To make the calculation more convenient, all of the experiments in this paper are using smartphone by right hand. If the experimenter uses his left hand, due to the symmetry of the coordinate axes, the region can be calculated and defined easily. Considering the different using-modes of smartphone, each of the three modes has its own threshold, as shown in Formula (11). *J* is flag number. 1 for Mode 1, 2 for Mode 2 and 3 for Mode 3. The thresholds in Formula (11) are statistical values, which are obtained by averaging the data of multiple experiments.

(11) $$\;\{\begin{array}{cc} J=1 & 70{}^{\circ}<\alpha <108{}^{\circ},\;14{}^{\circ}<\beta <59{}^{\circ}\\ J=2 & -12{}^{\circ}<\alpha <24{}^{\circ},\;14{}^{\circ}<\beta <59{}^{\circ}\\ J=3 & 108{}^{\circ}<\alpha <132{}^{\circ},\;78{}^{\circ}<\beta <100{}^{\circ} \end{array}$$

**Figure 6:**
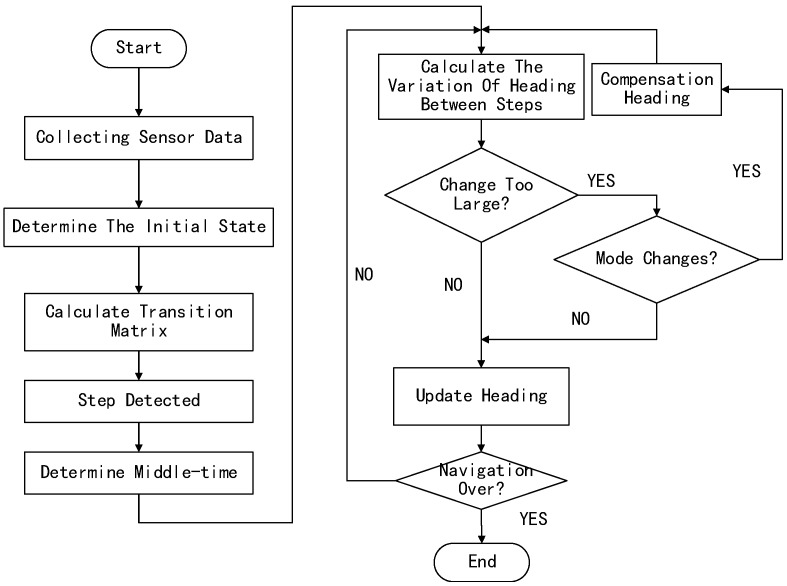
The General Process of GA Algorithm.

Based on the study of the relationship between three using-modes and the data of the smartphone, a new method to compensate the heading is proposed in this paper. The general process of this method is shown in Figure 6. A specific process has been divided into the four steps as mentioned below.

(1) Collecting the sensors’ data, and judging step moment to find out the Middle-time;
(2) Monitoring the change-value of the heading between two adjacent Middle-time;
(3) According to the change-value, to check the smartphone using-mode change;
(4) If there is using-mode change, compensate the heading value.

## 5. Experiment and Analysis

**Figure 7:**
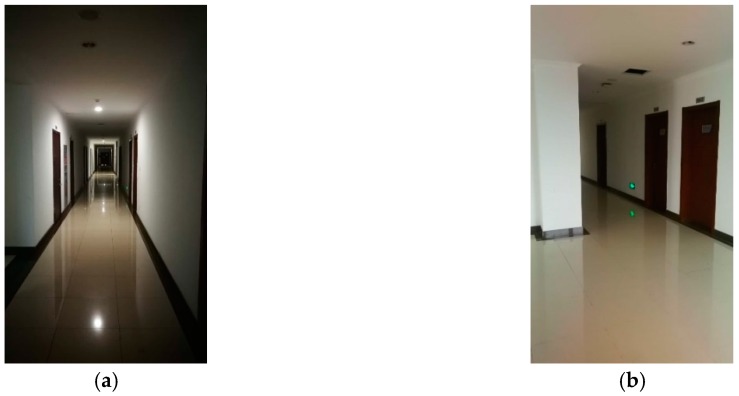
The Environment of Walking Experiments. (**a**) One of the corridors of experiment area; (**b**) one of the corners of the Experiment area.

In order to validate the mode discrimination method and heading compensation algorithm proposed in this paper, the researchers conducted the indoor walking experiments in the buildings of College of Automation Engineering (CAE) of NUAA, shown as in Figure 7. The data sampling frequency of the sensors is 50 Hz.

### 5.1. Accuracy Analysis of Smartphone Sensors

Allan variance method is often used to analyze the error components of accelerometers and gyroscopes. Before the experiments, the consumer-grade MEMS sensors in a smartphone (ONEPLUS A3000) are analyzed with Allan variance method. The variance can be expressed as the sum of the squares of the variances of the five noise sources:

(12) $$\;\sigma^{2}\left(\tau \right)=\frac{3Q^{2}}{\tau^{2}}+\frac{N^{2}}{\tau }+{\left(0.664B\right)}^{2}+\frac{K^{2}\tau }{3}+\frac{R^{2}\tau^{2}}{2}\;$$

In Formula (12), *N*, *K*, *R*, *B* and *Q* are Angle Random Walk (ARW), Rate Random Walk (RRW), Rate Ramp (RR), Bias Instability (BI), and Quantization Noise (QN), respectively. We calculate the ARW and BI to demonstrate the accuracy of smartphone sensors. The results of ARW and BI analysis are shown in Table 2 and Table 3. From the Table 2 and Table 3 we can see that the accuracy of the gyroscopes and accelerometers in the smartphone is very poor, and the body shaking will have a considerable impact on the heading of the smartphone navigation system.

### 5.2. Experiment Comparison of Step-Time and Middle-Time

In order to verify the algorithms mentioned in Section 3, two different experiments have been performed. The first one is walking straight for 52 m, and the second one is walking around the corridor of the CAE, a closed rectangle, about 41 m × 52 m. Since we did not use magnetic heading in this paper, we set the initial heading as 0 degree. In the straight walking experiment, the result is also the error angle relative to the actual heading. For the rectangular walking experiment, each turn angle is 90 degrees.

In this section, three algorithms have been applied to examine the results by using the same experiment data. They are traditional PDR algorithm, STS-ZUPT algorithm, and MTS-ZUPT algorithm. 

**Figure 8:**
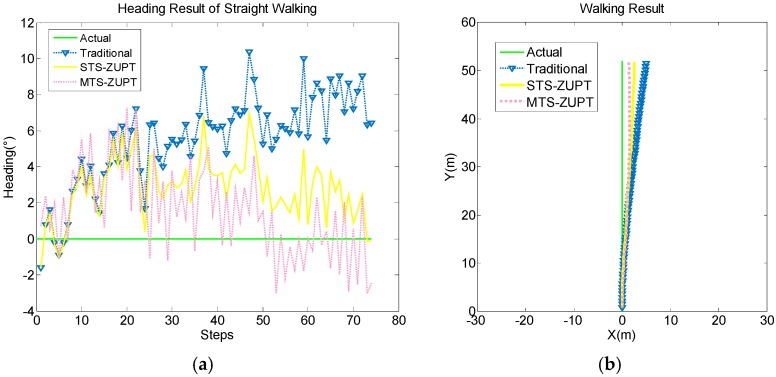
Straight Walking Result Comparison of MTS-ZUPT and STS-ZUPT. (**a**) Heading result; (**b**) Route result.

**Figure 9:**
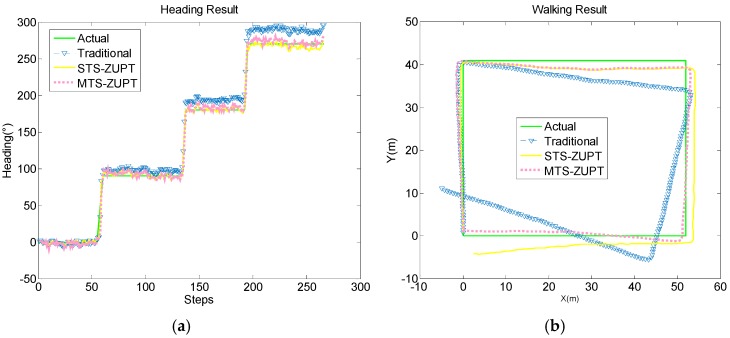
Rectangular Walking Result Comparison of STS-ZUPT and MTS-ZUPT. (**a**) Heading result; (**b**) Route result.

In the Figure 8 and Figure 9, the green line is the actual walking reference, the yellow line is the STS-ZUPT result, the red dotted line is the MTS-ZUPT result, while the blue lower triangle is the traditional PDR result.

From above four Figures, we can see that the S-ZUPT algorithm has the ability to restrain the heading error divergence for STS-ZUPT and MTS-ZUPT, and MTS-ZUPT has better performance.

In Figure 8b, the final positioning point of MTS-ZUPT is (1.369, 51.73), the final positioning point of STS-ZUPT is (2.512, 51.72), while the final positioning point of traditional PDR is (4.816, 51.51). positioning accuracy of MTS-ZUPT is 44.8% better than that of STS-ZUPT, and 71.2% better than that of traditional PDR algorithm.

And in the Figure 9b, the final positioning point of MTS-ZUPT, STS-ZUPT and traditional PDR are (0.030, 1.341), (2.347, −4.224), (−4.97, 11), respectively. The positioning accuracy of MTS-ZUPT is 72.2% better than that of STS-ZUPT, and 88.8% better than that of traditional PDR. We can see that the result of MTS-ZUPT is much closer to the actual walking route. At the turning points, MTS-ZUPT still gives a result close to 90 degrees. MTS-ZUPT algorithm can reduce the walking error, which is only about 0.72% of whole walking distance.

The above results show that the MTS-ZUPT algorithm proposed in this paper can improve the heading accuracy and restrain the error divergence with only MEMS IMU information.

### 5.3. Experiment of GA and MTS-ZUPT

**Figure 10:**
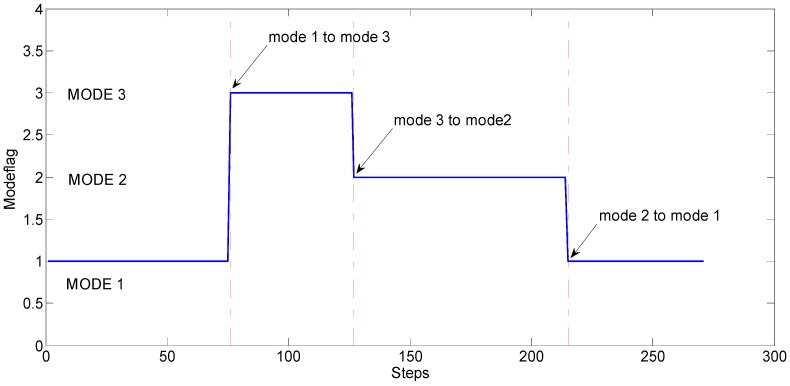
Judgment of Smartphone Using-mode.

In this section, the experiment has been carried out in the corridor of CAE. The walking route is similar as former experiment route, a closed rectangular. The three using-modes mentioned in Figure 4 are demonstrated when walking, and the using-mode changing order is: from mode 1 to mode 3, then to mode 2, and back to mode 1. The traditional PDR algorithm, PDR/GA algorithm, MTS-ZUPT algorithm and the MTS-ZUPT/ GA algorithm are applied to deal with the collected data. Figure 10 shows the using-mode judgement result of smartphone by GA algorithm. The red point-dotted lines represent the moment when the using-mode changes. From Figure 10, we can see that three using-modes are distinguished successfully with 100% accuracy.

**Figure 11:**
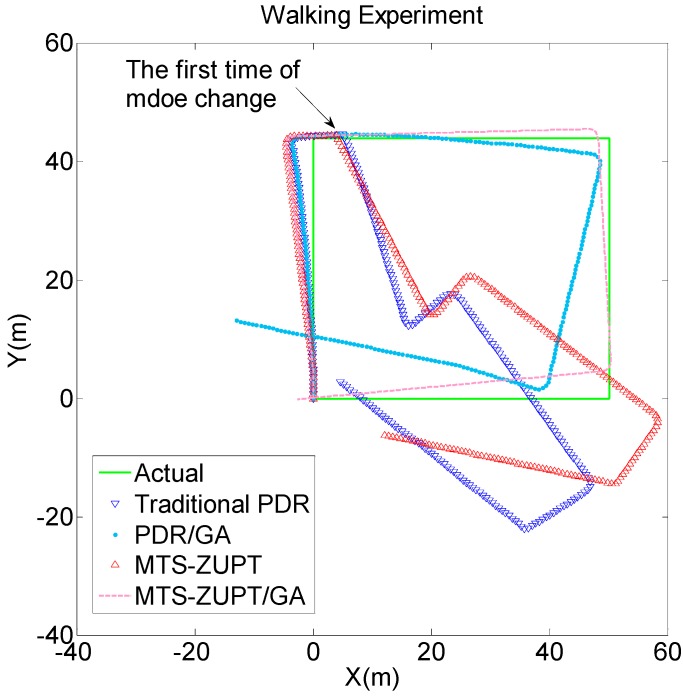
Walking Experiments Comparison.

The walking track results are shown in Figure 11. The green solid line represents the actual walking trajectory; the blue lower triangle and the red upper triangle represent the PDR algorithm and the MTS-ZUPT algorithm results. The blue dotted line represents PDR/GA algorithm result, the red dotted line represents MTS-ZUPT/GA algorithm result.

**Figure 12:**
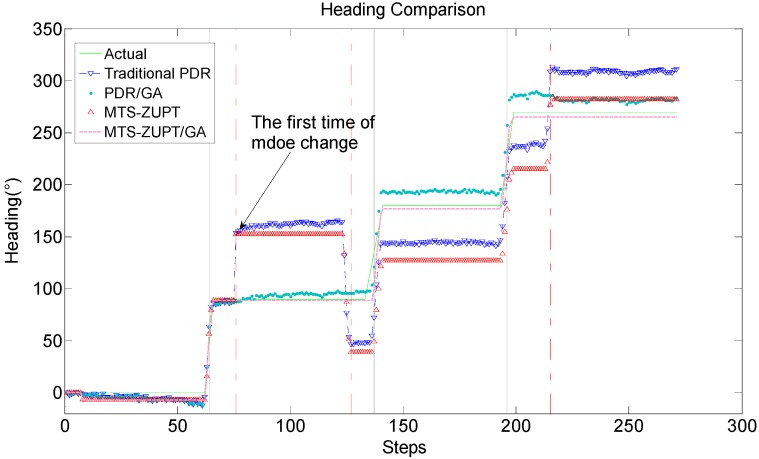
Heading Comparison.

Figure 12 shows the heading value curves of the four algorithms. Among them, the gray solid lines represent the turning moment of the experimenter during walking, and the red point-dotted lines are the using-mode changing moments. It can be clearly seen from Figure 12 that the GA algorithm does not affect the change of heading when the pedestrian normal walks and turns. However, once the using-mode has been changed, the algorithms without GA will output the wrong heading angles. With the help of GA algorithm, the navigation system can compensate the heading angle by judging the using-mode. Moreover, the heading result obtained by the MTS-ZUPT/GA algorithm is better than the heading result of the traditional PDR/GA algorithm.

It can be seen from Figure 11 and Figure 12 that the solution result without GA has a large deviation in the walking trajectory after the first using-mode change. After three times of the using-mode changes, the walking trajectory has completely deviated from the actual walking route. For two algorithms aided by GA algorithm, the walking trajectory is very close to the actual route. We can see that the GA algorithm proposed in this paper can effectively judge the using-mode change of the smartphone, and can compensate the corresponding heading values. The final positioning point of the MTS-ZUPT/GA algorithm is (−2.747, 0.1757), and its distance error is 2.75 m, which is 1.39% for the walking distance of 198 m. Compared with the PDR/GA algorithm result (−13.03, 13.16), the accuracy of the final positioning result has been increased by 80%. From Figure 11 and Figure 12, we can see that the MTS-ZUPT/GA designed in this paper can considerably improve the navigation accuracy and restrain the error divergence of inertial navigation.

**Figure 13:**
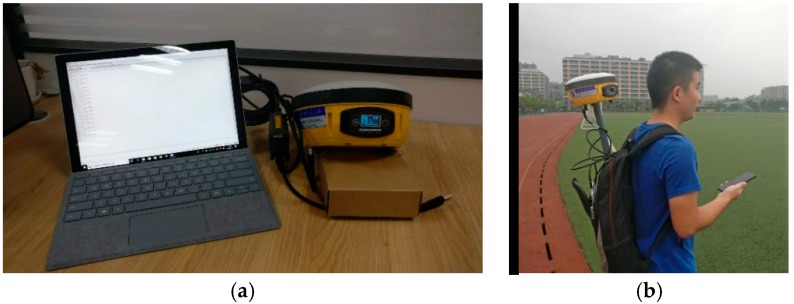
DGPS Device and the Wear Mode of Playground Experiment. (**a**) The DGPS device and the data storage device (Surface tablet PC); (**b**) The specific equipment for playground experiment.

To verify the MTS-ZUPT/GA algorithm, we completed another experiment with another smartphone (MI MIX2) on one of the playgrounds in NUAA. In this outdoor environment, the positioning information from a DGPS (Differential Global Positioning System) device with centimeter-level positioning accuracy has been used as a reference. The preparatory work of the playground experiment can be found in Figure 13. The DGPS device has been connected to the Surface tablet PC as shown in Figure 13a. The experiment performer is holding the smartphone in his right hand, and carrying the DGPS and Surface tablet PC in his backpack, as shown in Figure 13b.

**Figure 14:**
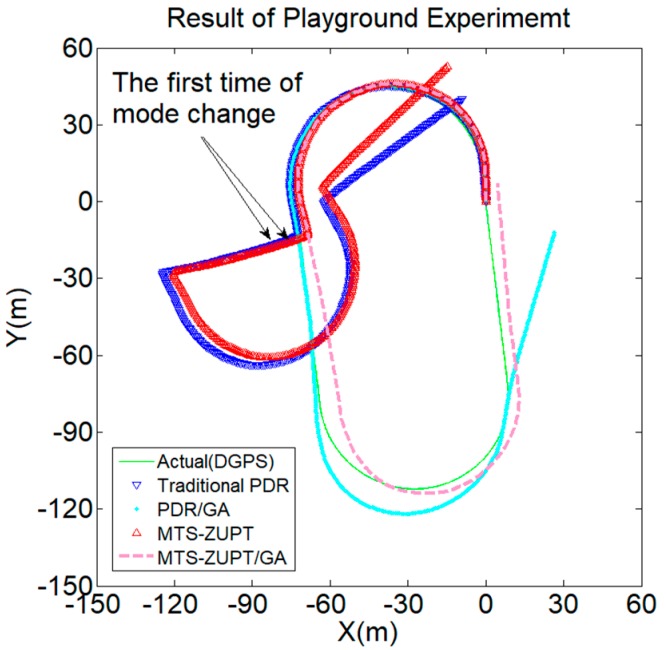
Result of Playground Experiment.

The experiment result is shown in Figure 14. The green line is the positioning trajectory of DGPS. During the whole experiment, there are three using-mode changes, the first moment using-mode change point is marked in the Figure 14. We can find that this experiment result is very similar to the result in the corridor of CAE. Overall, the differences between the result of MTS-ZUPT/GA algorithm and the positioning trajectory of DGPS is minimal. In this situation, both the start and end points are (0, 0). The final end point of MTS-ZUPT/GA algorithm is (4.70, 6.92), the error is 2.0% for the walking distance of 400 m. The final end point of PDR/GA algorithm is (26.44, −12.2). The experimental results show the superiority of the MTS-ZUPT/GA algorithm compared to the other algorithms. 

## 6. Conclusions

To avoid the heading interference caused by the magnetic disturbance, an autonomous inertial heading correction algorithm without magnetic field was put forward. From the results of MTS-ZUPT and STS-ZUPT, we can see that the MTS-ZUPT algorithm is most effective for improving the PDR performance, and it is more effective than STS-ZUPT algorithm in restraining error divergence. From the results of MTS-ZUPT/GA and STS-ZUPT/GA algorithm, it can be seen that the GA algorithm can realize the using-mode judgment of the smartphone and compensate the heading error.

The experimental results indicate that the MTS-ZUPT/GA algorithm proposed in this paper has a positioning error of 1.4% to 2.0% of the walking distance in different using-mode experiments. It can effectively reduce the using-mode error, restrain the heading divergence, enhance the navigation performance of smartphone.

## Figures and Tables

**Table 1:** Experiment Results of Step Detection.

No.	Real	Algorithm	Accuracy
1	60	59	98.3%
2	100	99	99%
3	150	148	98.6%
4	200	200	100%

**Table 2:** Results of Gyroscope Allan Variance.

Gyroscope	$\;N\left({}^{\circ}/h^{1/2}\right)\;$	B(°/h)
X-axis	0.4482	23.6184
Y-axis	0.5655	9.6157
Z-axis	0.4238	10.2627

**Table 3:** Results of Accelerometer Allan Variance.

Accelerometer	$\;N\left(m/s^{3/2}\right)\;$	$\;B\left(m/s^{2}\right)\;$
X-axis	$\;2.0883\times 10^{-5}$	$\;2.53\times 10^{-4}$
Y-axis	$\;2.0171\times 10^{-5}$	$\;4.33\times 10^{-4}$
Z-axis	$\;2.2924\times 10^{-5}$	$\;5.68\times 10^{-4}$
